# Machine learning developed a CD8^+^ exhausted T cells signature for predicting prognosis, immune infiltration and drug sensitivity in ovarian cancer

**DOI:** 10.1038/s41598-024-55919-4

**Published:** 2024-03-09

**Authors:** Rujun Chen, Yicai Zheng, Chen Fei, Jun Ye, He Fei

**Affiliations:** 1 Department of Obstetrics and Gynecology, Shanghai Fifth People’s Hospital, Fudan University, Shanghai, 200240 China; 2 Department of Stomatology,Shanghai Fifth People’s Hospital, Fudan University, Shanghai, 200240 China; 3 Shanghai Jiao Tong University, Shanghai, 200240 China

**Keywords:** CD8^+^ T_ex_, Machine learning, Ovarian cancer, Prognostic signature, Immunotherapy, Urological cancer, Machine learning, Computational biology and bioinformatics, Biomarkers

## Abstract

CD8^+^ exhausted T cells (CD8^+^ T_ex_) played a vital role in the progression and therapeutic response of cancer. However, few studies have fully clarified the characters of CD8^+^ T_ex_ related genes in ovarian cancer (OC). The CD8^+^ T_ex_ related prognostic signature (TRPS) was constructed with integrative machine learning procedure including 10 methods using TCGA, GSE14764, GSE26193, GSE26712, GSE63885 and GSE140082 dataset. Several immunotherapy benefits indicators, including Tumor Immune Dysfunction and Exclusion (TIDE) score, immunophenoscore (IPS), TMB score and tumor escape score, were used to explore performance of TRPS in predicting immunotherapy benefits of OC. The TRPS constructed by Enet (alpha = 0.3) method acted as an independent risk factor for OC and showed stable and powerful performance in predicting clinical outcome of patients. The C-index of the TRPS was higher than that of tumor grade, clinical stage, and many developed signatures. Low TRPS score indicated a higher level of CD8^+^ T cell, B cell, macrophage M1, and NK cells, representing a relative immunoactivated ecosystem in OC. OC patients with low risk score had a higher PD1&CTLA4 immunophenoscore, higher TMB score, lower TIDE score and lower tumor escape score, suggesting a better immunotherapy response. Moreover, higher TRPS score indicated a higher score of cancer-related hallmarks, including angiogenesis, EMT, hypoxia, glycolysis, and notch signaling. Vitro experiment showed that ARL6IP5 was downregulated in OC tissues and inhibited tumor cell proliferation. The current study constructed a novel TRPS for OC, which could serve as an indicator for predicting the prognosis, immune infiltration and immunotherapy benefits for OC patients.

## Introduction

Ovarian cancer (OC) is the leading cause of gynecological cancer death and the fifth most common cause of cancer death in women in the USA^1^. A total of 19,880 cases are estimated to be initially diagnosed with OC and 12,810 patients die from this malignancy in the USA in 2022^2^. Despite many management approaches have been used for the treatment of ovarian cancer, including surgery, chemotherapy, and endocrine therapy, the clinical outcome of OC cases are still poor, with the 5-year survival rate less than 50%^1^. In addition to TNM staging system, there are few clinical markers for predicting the prognosis of OC patients. High recurrence and drug resistance remain the main reasons for the poor clinical outcomes for ovarian cancer patients^3,4^. Due to lack of typical clinical symptoms in the early stage, many patients have advanced disease or distant metastasis by the time ovarian cancer is diagnosed. Recent study showed that immunotherapy could be a promising modality for many malignancies, especially for advanced malignancies^5^. However, the evidences about OC response to immunotherapy and biomarkers for predicting the immunotherapy response are limited.

Dynamic interactions between OC and tumor microenvironment (TME) are vital for the heterogeneity and therapeutic response of OC. Previous study has highlighted the critical functions of tumor-infiltrating lymphocyte (TILs) in the progression and therapeutic response of OC^6^. Different subtypes of TILs have different functions. CD8^+^ exhausted T cells (CD8^+^ T_ex_) are a subtype of TILs characterized by weak ability in clearing a pathogenic threat, blockading surface co-inhibitory receptors, hypo-response to anti-tumor immunotherapies^7^. CD8^+^ T_ex_ persist in the TME and interact with tumor cell and other subtype of TILs, which can affect the progression and therapeutic response of cancer. However, few studies have fully clarified the characters of CD8^+^ T_ex_ related genes (TRGs) in OC.

**Figure 1:**
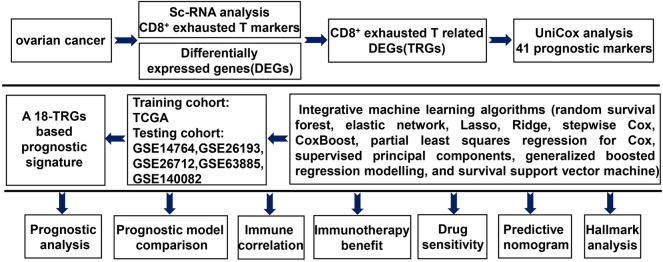
Workflow of our study.

As shown in Fig. 1, we developed an 18-gene basedCD8^+^ T_ex_ related prognostic signature (TRPS) for OC using TCGA, GSE14764, GSE26193, GSE26712, GSE63885 and GSE140082 datasets. We then explored the correlation between TRPS and the prognosis, immune infiltration, immunotherapy benefits and signaling pathway in OC, offering insights into prognosis prediction and immune landscape in OC.

## Materials and methods

### Datasets sources

Single cell expression data of OC tissues was obtained from GSE184880 dataset (n = 7). Bulk RNA-seq data of OC cases and normal ovarian cases were obtained from TCGA database (n = 374) and GTEx database (n = 64), respectively. Five GEO datasets, including GSE14764 (n = 80), GSE26193 (n = 107), GSE26712 (n = 185), GSE63885 (n = 75) and GSE140082 (n = 380), were used for TRPS validation. To explore the role of TRPS in predicting immunotherapy benefit, we also downloaded two immune therapy datasets, including IMvigor210 dataset (n = 298) and GSE91061 dataset (n = 98). These two immunotherapy datasets included clinical information about the patients treated with anti-PD-L1 and anti-CTLA4 agents.

### scRNA-seq analysis

scRNA-seq data was used for cell marker identification. Further detail was shown in Supplementary methods and results. The “FindAllMarkers” function of the Seurat package was used for cell marker identification with the minimum cell population fraction in either of the two populations of 0.25. TRGs were identified as the marker genes of CD8^+^ T_ex_ corresponding to clusters.

### Machine learning algorithms developed a TRPS

Differentially expressed genes (DEGs) in OC were identified used “limma” package using |LogFC| ≥ 1.5 as the cutoff. Univariate cox analysis was performed to identify potential biomarkers. Prognostic biomarkers were then submitted to integrative analysis procedure for developing a TRPS. Further detail was shown in Supplementary methods and results. We then calculated the Harrell’s concordance index (C-index) of all models in training (TCGA) and testing (GEO) cohort based on the expression of candidate genes and corresponding coefficient. The prognostic TRPS with the highest average C-index was regarded as the optimal prognostic signature.

### Evaluation of the performance of TRPS

Using the “surv_cutpoint” function of the R package “survminer”, we obtained the best cut-off and separated OC cases into low and high TRPS score (risk score) groups. As many prognostic signatures have been developed for OC, we then collected 45 prognostic signatures randomly (Supplementary Table 1) and calculated their C-index using “rms” package, with which we could compare their performance in predicting the clinical outcome of OC patients. Univariate and multivariate cox analysis were conducted to explore the risk factor for the overall survival rate of OC patients. Using “nomogramEx” R package, we then developed a predicting nomogram.

### Immune infiltration analysis

Immunedeconv was used to explore the correlation between risk score and immune cells (Supplementary methods and results). To evaluate ESTIMATE score of each OC case, we then applied “estimate” R package^8^. Hallmark gene set was downloaded from Molecular Signatures Database (MSigDB). ssGESA was conducted to detect the score of hallmark gene set, immune cells and related functions of each OC case.

### Immunotherapy benefit and drug sensitivity

Several immunotherapy benefits indicators, including the Tumor Immune Dysfunction and Exclusion (TIDE) score, immunophenoscore (IPS), tumor mutation burden (TMB) score and tumor escape score, were used to explore performance of TRPS in predicting immunotherapy benefits of OC. From the Cancer Immunome Atlas (https://tcia.at/home), we downloaded the IPS of ovarian cancer cases. TIDE score and T cells exclusion scores of ovarian cancer cases were downloaded from TIDE (http://tide.dfci.harvard.edu). The oncoPredict R package was used to calculate the IC50 of drugs in each OC case using the data of Genomics of Drug Sensitivity in Cancer (https://www.cancerrxgene.org/).

### Cell lines and overexpression of ARL6IP5

Normal ovarian cell line (Hs823.Tc) and OC cell lines (ES-2, OV90, TOV21G, CaOV-3, SKOV3, TOV112D) were purchased from Shanghai Institute of Biochemistry and Cell Biology (Shanghai, China). Cells were maintained in circumstances containing 5% CO_2_ and 95% saturated humidity at 37 °C using respective ATCC recommended medium. Fetal bovine serum (FBS; Gibco) and 1% penicillin–streptomycin (Sigma-Aldrich, St. Louis, USA) were added to the medium. A pcDNA3.1 plasmid encoding the human ARL6IP5 and empty vector was purchased from GenScript (Nanjing, China). Lipofectamine 3000 transfection reagent (Invitrogen, Thermo Fisher Scientific) was used to transfect the plasmid into OV cell lines based on the manufacturer’s instructions.

### RT-qPCR and proliferation assay

Using TRIzol (Takara Bio, Dalian, China), we extracted RNA from cells, which were reversely transcribed into cDNA using an oligo (dT) primer subsequently. Based on the ABI 7900HT detection system (Thermo Fisher Scientific Inc.), we then performed RT-qPCR with SYBR Premix Ex Taq (Takara Bio). Gene expression levels were normalized to the endogenous GAPDH. For proliferation assay, OV cell lines were plated in 96-well plates (5000 cells/well in triplicates). Cell Counting kit-8 (CCK-8; Beyotime) was added to cells at indicated times. Proliferation index was calculated as the ratio of OD value at the indicated time/OD value of the input cells.

### Statistical analysis

Statistical analyses were performed with R software (version 4.2.1). The difference between continuous variables was evaluated with Wilcoxon rank-sum test or Student *t* test. Pearson’s or Spearman's rank correlation analysis was conducted to analyze the correlations between two continuous variables. The two-sided log-rank test was used to test the difference in different Kaplan–Meier survival curve.

## Results

### Identification of TRGs and their prognostic value

**Figure 2:**
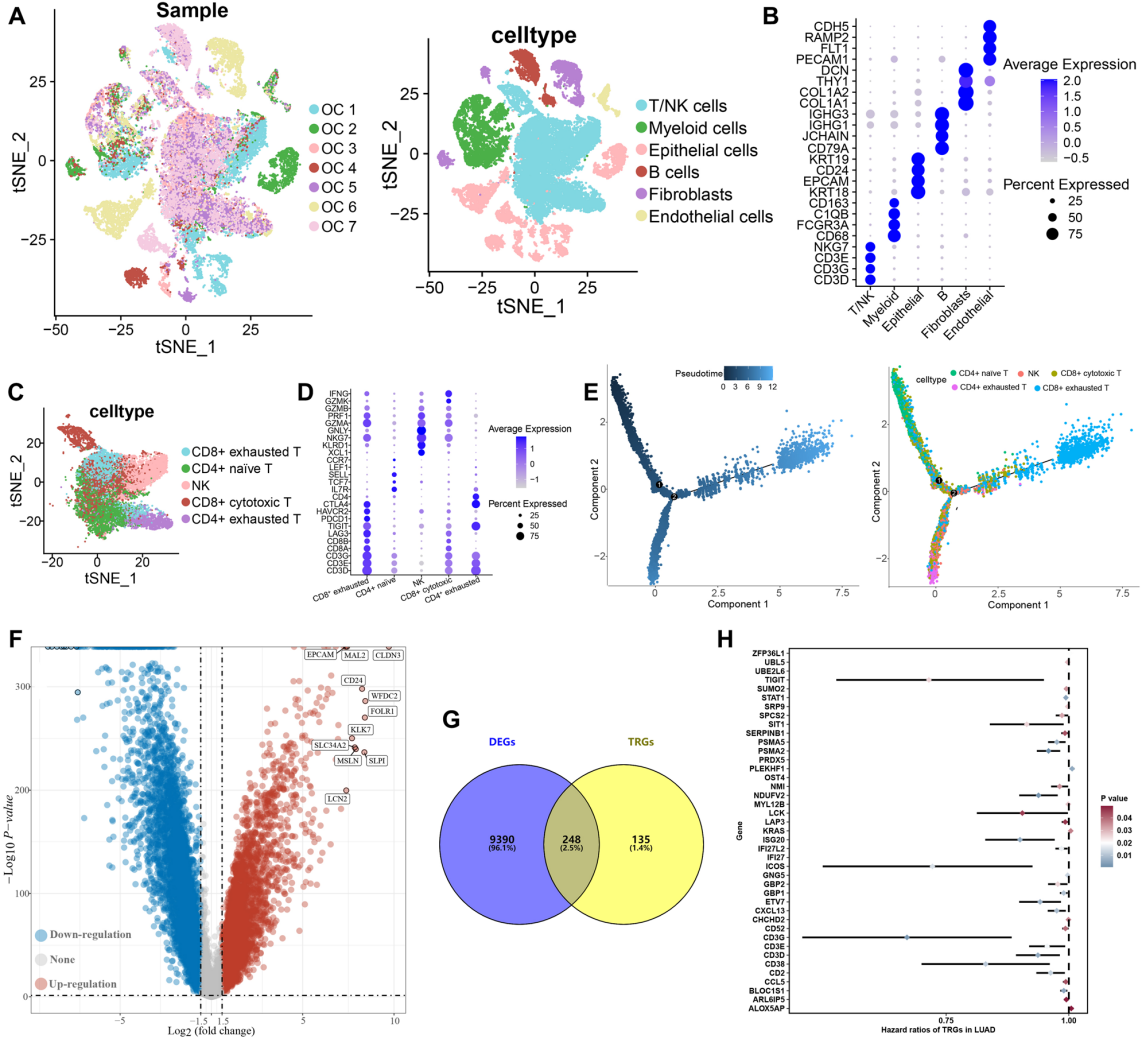
Identification of TRGs and their prognostic value. (**A**) t-SNE plot showing the identified cell types of from 7 ovarian cancer sample. (**B**) Dotplot showing average expression levels of cell marker. (**C**,**D**) SNE plot of sub-cell types of T cells and dotplot of expression pattern of cell markers. (**E**) Developmental trajectory of T cells inferred by monocle, colored by pseudotime and cell subtype. (**F**) Volcano plot showing DEGs in ovarian cancer. (**G**) Overlap between DEGs and TRGs. (**H**) Potential biomarkers identified by univariate cox analysis.

From the data obtained from the single-cell RNA-seq analyses of OC tissue (GSE184880 dataset), we identified six major types of cells, including T/NK cells, myeloid cells, Epithelial cells, Fibroblasts, B cells and endothelial cells (Fig. 2A). Figure 2B showed the expression of cell markers. We then extracted T/NK cells for further analysis. As result, T/NK cells could be re-clustered into CD8^+^ cytotoxic T, CD8^+^ exhausted T, NK, CD4^+^ exhausted T and CD4^+^ naïve T based on expression pattern of cell markers (Fig. 2C,D). Development trajectory analyses of T/NK cells unveiled that CD4^+^ naïve T, CD8^+^ cytotoxic T, and NK were enriched in initial differentiation phase while CD4^+^ exhausted T and CD8^+^ exhausted T were enriched in terminal differentiation phase (Fig. 2E). Based on the “FindAllMarkers” function of the Seurat package, we identified 384 TRGs. Compared with normal tissues, we obtained 9638 DEGs in OC tissues (Fig. 2F), including 248 TRGs (Fig. 2G) in TCGA dataset. Among these differentially expressed TRGs, a total of 41 genes were significantly associated with the prognosis of OC patients in TCGA dataset (Fig. 2H, P < 0.05).

### Integrative machine learning algorithms developed a TRPS

**Figure 3:**
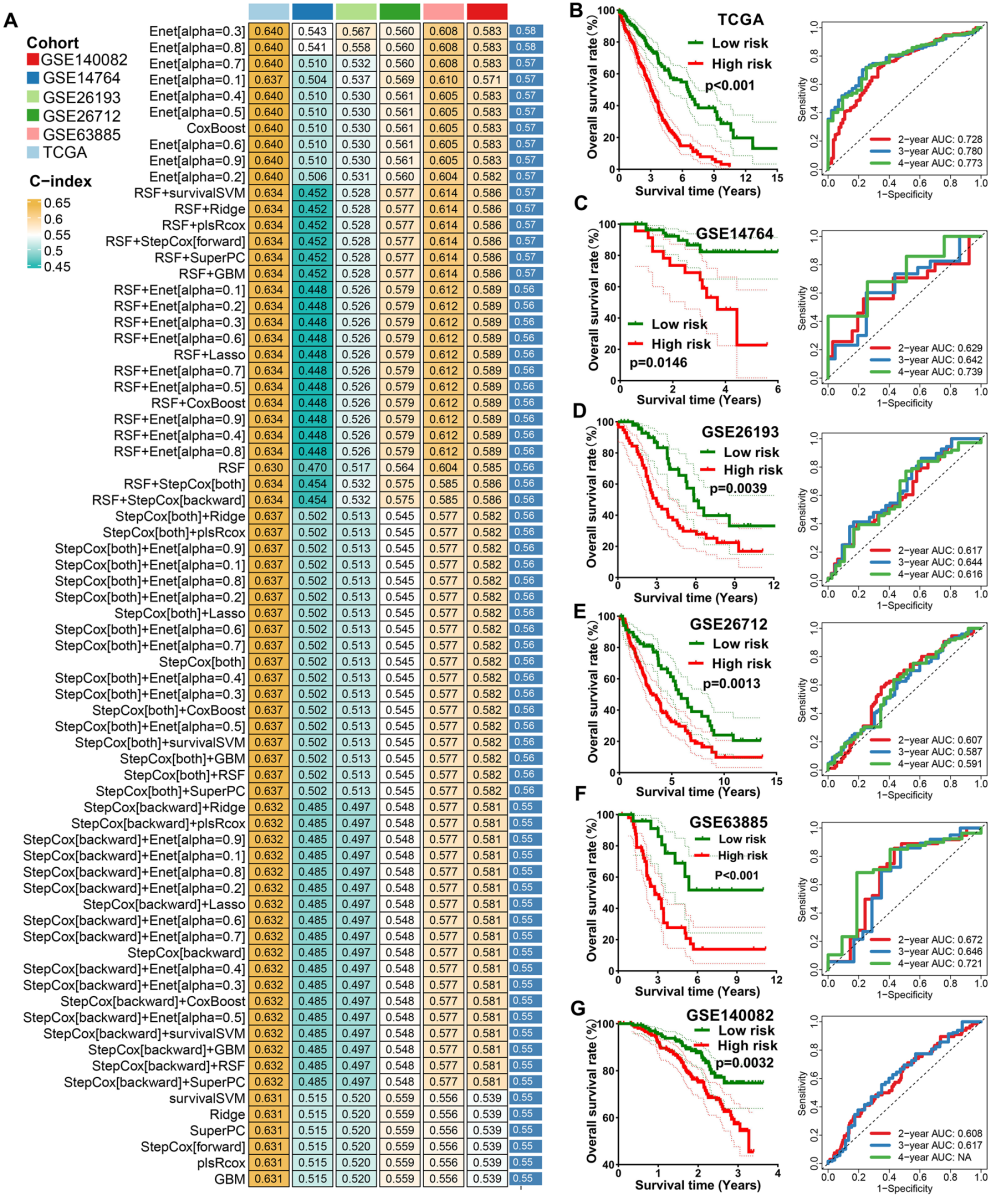
Identification of TRPS by machine learning. (**A**) The C-index of 101 kinds prognostic models constructed by 10 machine learning algorithms in training and testing cohort. (**B**–**G**) The survival curve of ovarian cancer patients with different TRPS score and their corresponding ROC curve in TCGA, GSE14764, GSE26193, GSE26172, GSE63885 and GSE140082 cohort.

These 41 potential prognostic biomarkers were submitted to an integrative machine learning procedure including 10 methods, with which we developed a stable TRPS. As a result, we obtained a total of 101 kinds of prognostic models and their C-index in training and testing cohorts were shown in Fig. 3A. The data suggested that the prognostic signature constructed by Enet (alpha = 0.3) method was considered as the optimal TRPS with a highest average C-index of 0.58 (Fig. 3A). The optimal TRPS was developed by 18 TRGs. The formula of the risk score was shown in Supplementary methods and results. Using the best cut-off value, we then divided into ovarian cancer cases into high and low TRPS score. As expected, OC patients with high risk score had a poor OS rate in TCGA cohort (P < 0.001), GSE14764 cohort (P = 0.0146), GSE26193 cohort (P = 0.0039), GSE26712 cohort (P = 0.0013), GSE63885 cohort (P < 0.001) and GSE140082 (P = 0.0032) cohort (Fig. 3B–G), with the AUCs of 2-, 3-, and 4-year being 0.728, 0.783, and 0.773 in TCGA cohort; 0.629, 0.642, and 0.739 in GSE14764 cohort; 0.617, 0.644, and 0.616 in GSE26193 cohort; 0.607, 0.587, and 0.591 in GSE26712 cohort, 0.672, 0.646 and 0.721 in GSE63885 cohort, 0.608 and 0.617 in GSE140082 cohort, respectively (Fig. 3B–G).

### Evaluation of the performance of TRPS

**Figure 4:**
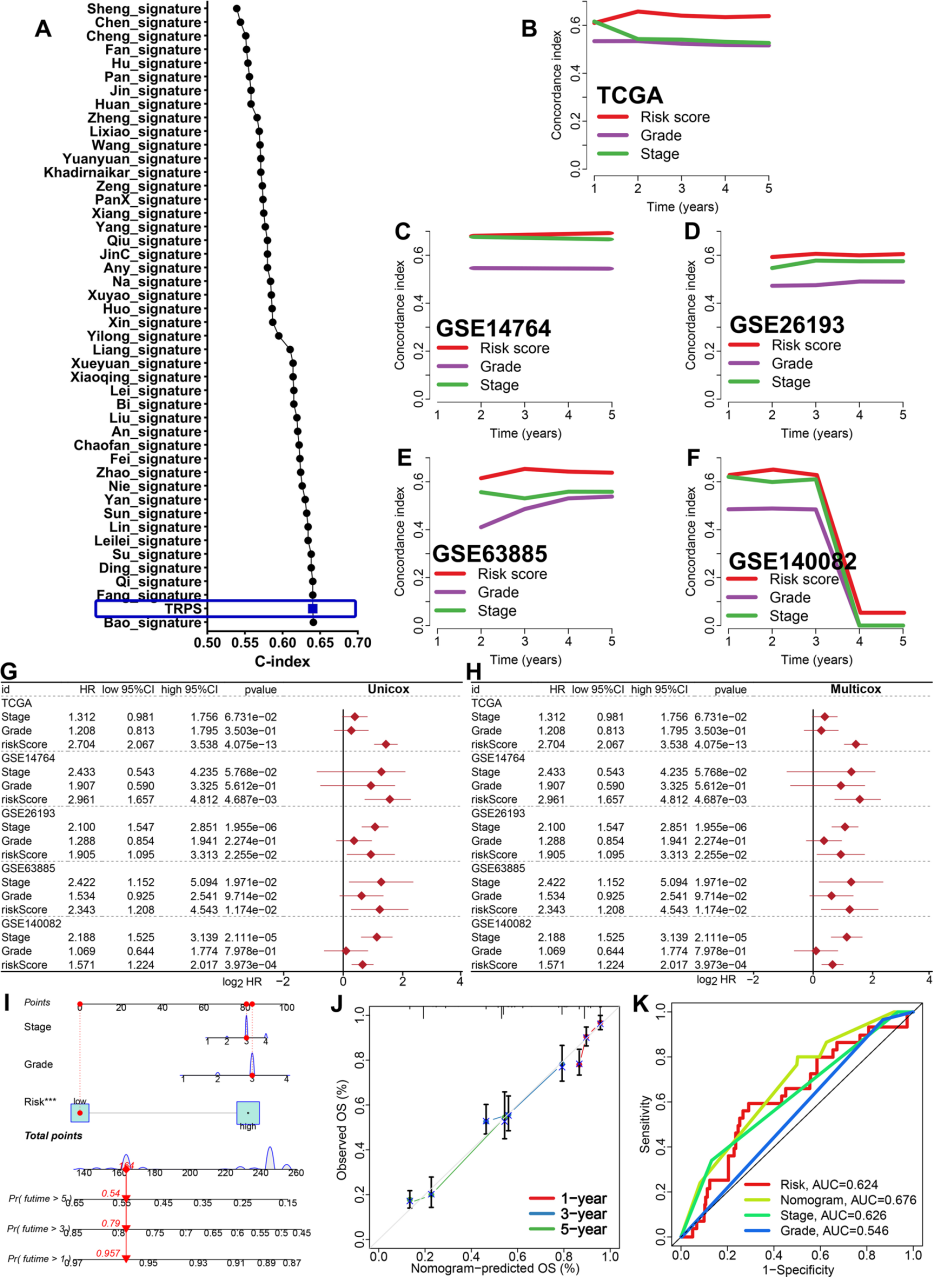
Evaluation the performance of TRPS in predicting prognosis of OC patients. (**A**) C-index of TRPS and other 45 established signatures in predicting the prognosis of OC patients. (**B**–**F**) The C-index of TRPS, tumor grade and clinical stage in predicting prognosis of OC patients in TCGA, GSE14764, GSE26193, GSE63885 and GSE140082 cohort. (**G**,**H**) Univariate and multivariate cox regression analysis considering grade, stage and TRPS in training and testing cohort. (**I**,**J**) Predictive nomogram and calibration evaluating the 1-y, 3-y and 5-y overall survival rate of OC patients. (**K**) ROC curve evaluated the performance of nomogram in predicting prognosis of OC patients.

To compare the performance of TRPS with other prognostic signatures in predicting the OS rate of OC cases, we randomly collected 45 OC-related prognostic signatures (Supplementary Table 1) and calculated their C-index. As a result, the C-index of TRPS was higher than most of these prognostic signatures in TCGA dataset (Fig. 4A). Moreover, the C-index of TRPS was higher than that of tumor grade and clinical stage in training and testing cohorts (Fig. 4B–F). These evidences suggested that the predictive value of TRPS in predicting the clinical outcome of OC patients was higher than most of signatures and clinical characters. However, we could not evaluate the predictive value of TRPS in predicting the OS rate of OC patients in GSE26712 cohort due to the missing data of tumor grade and clinical stage. Based on the result of univariate and multivariate cox regression analysis, TRPS served as an independent risk factor for the clinical outcome of OC patients in TCGA, GSE14764, GSE26193, GSE63885 and GSE140082 cohort (Fig. 4G,H, all P < 0.05). To predict the 1-year, 3-year and 5-year OS rate of OC patients, we then constructed a nomogram based on TRPS, clinical stage and tumor grade using TCGA dataset (Fig. 4I). The comparison between the predicted curve and the ideal curve showed a high coincidence in TCGA dataset (Fig. 4J). Compared with TPRS, clinical stage and tumor grade, the AUC of nomogram were higher in TCGA dataset (Fig. 4K).

### The distinct immune microenvironment in OC patients with different TRPS score

**Figure 5:**
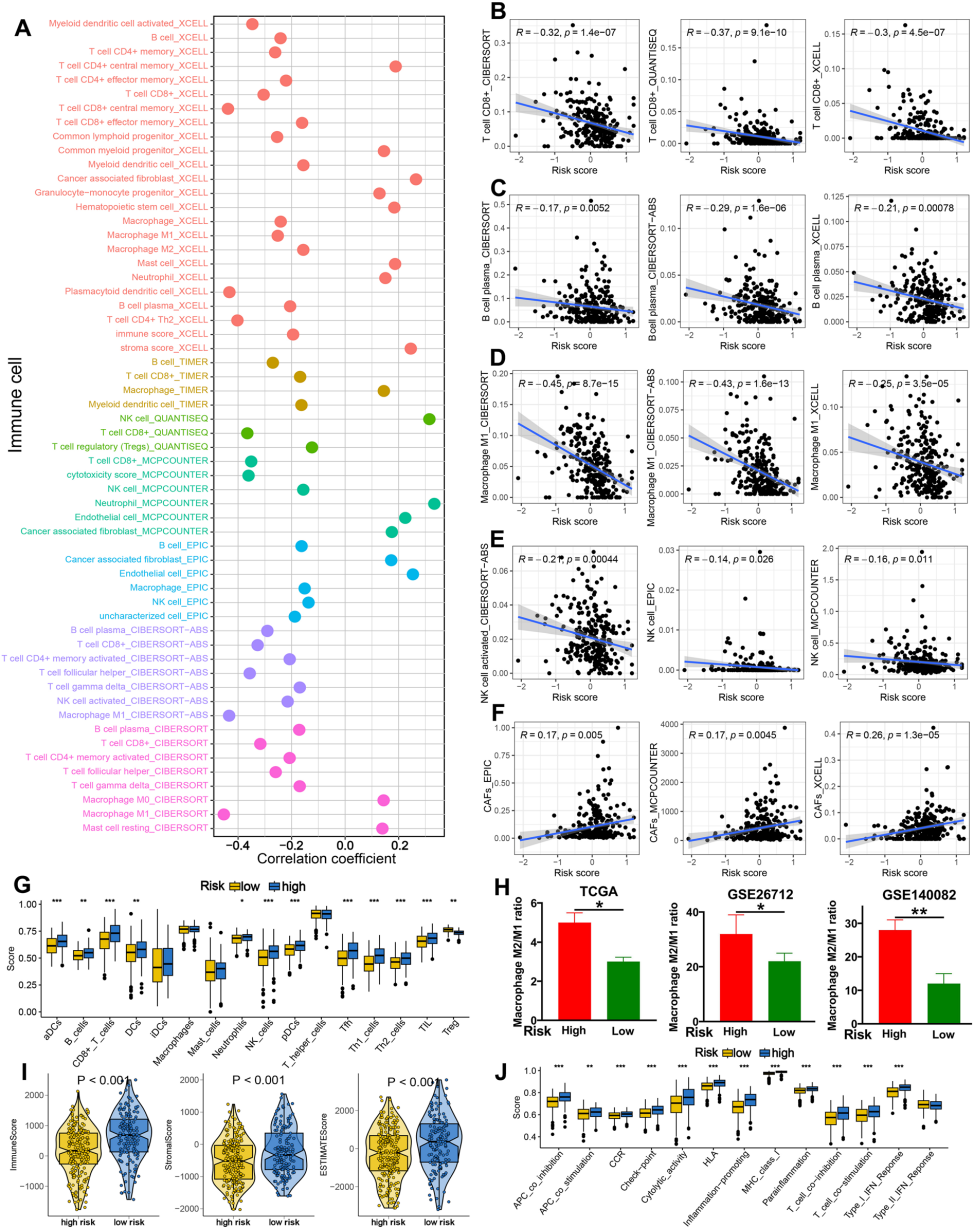
Correlation between immune microenvironment and TRPS in OC. (**A**) Seven state-of-the-art algorithms evaluating the correlation between TRPS and immune cell infiltration in OC. (**B**–**F**) The correlation between TRPS and the abundance of CD8^+^ T cells, plasma cells, macrophage M1 and CAFs. (**G**) The level of immune cells in different TRPS score group based on ssGSEA analysis. (**H**) The macrophage M2/M1 ratio in different TRPS score group in TCGA, GSE26712 and GSE140082 dataset. (**I**,**J**) The stromal score, immune score, ESTIMAE score and immune-related functions score in different TRPS score group. *P < 0.05, **P < 0.01, ***P < 0.001.

As shown in Fig. 5A, TRPS showed significant correlation with the abundance of immune cells in TCGA dataset (all P < 0.05). More specifically, TRPS showed a negative correlation with immuno-activated cell infiltration, such as CD8^+^ T cells, plasma cells, macrophage M1 and NK cells in TCGA dataset (Fig. 5B–E, all P < 0.05). Interestingly, higher risk score indicated a higher level of cancer-related fibroblasts in TCGA dataset (Fig. 5F). Similar results were obtained in ssGSEA analysis, suggesting a higher abundance of immuno-activated cells in low risk score group, including aDCs, B cells, CD8^+^ T cells, Neutrophils, NK cells, Tfh and TIL in TCGA dataset (Fig. 5G, all P < 0.05). Previous studies showed that macrophage M2/M1 polarization played a vital role in the progression of cancer^9,10^. Our study showed that OC patients with high risk score had a higher macrophage M2/M1 polarization in TCGA, GSE26712, and GSE140082 cohort (Fig. 5H, all P < 0.05). Further analysis suggested a higher stromal score, immune score and ESTIMAE score in low risk score group in TCGA dataset (Fig. 5I, all P < 0.001). Moreover, higher risk score indicated a higher APC co-stimulation score, CCR score, cytolytic activity score, para-inflammation promoting score, parainflammation and T cell co-stimulation score in TCGA dataset (Fig. 5J).

### TRPS could predict the therapy benefits of OC patients

**Figure 6:**
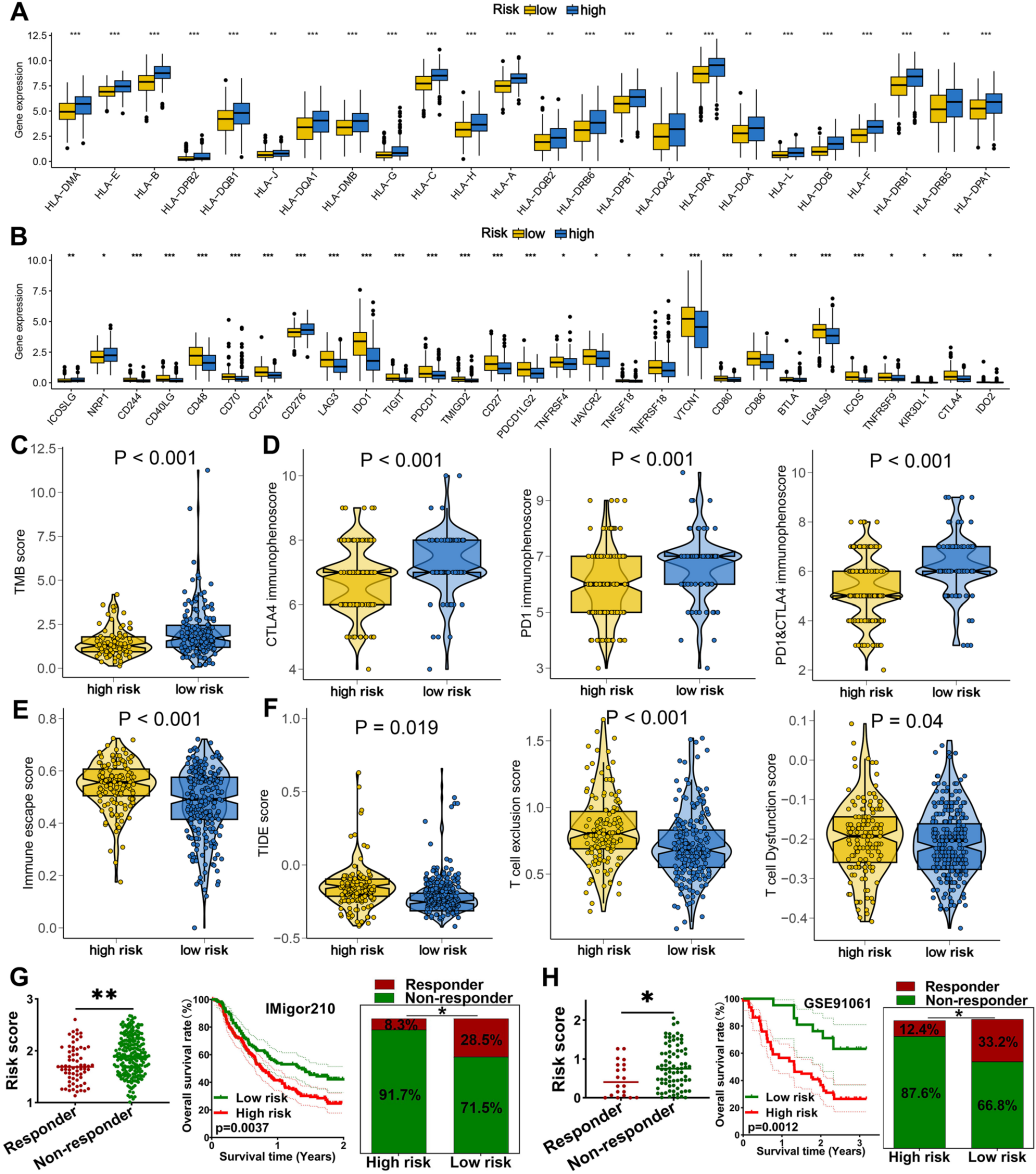
TRPS as an indicator for immunotherapy response in OC. (**A**,**B**) The level of HLA-related genes and immune checkpoints in different TRPS score group. (**B**–**F**) The TMB score, immunophenoscore, immune escape score and TIDE, T cell dysfunction and exclusion score in different TRPS score group. (**G**,**H**) The overall rate and immunotherapy response rate in patients with high and low risk score in GSE91061 and IMvigor210 cohort. *P < 0.05, **P < 0.01, ***P < 0.001.

**Figure 7:**
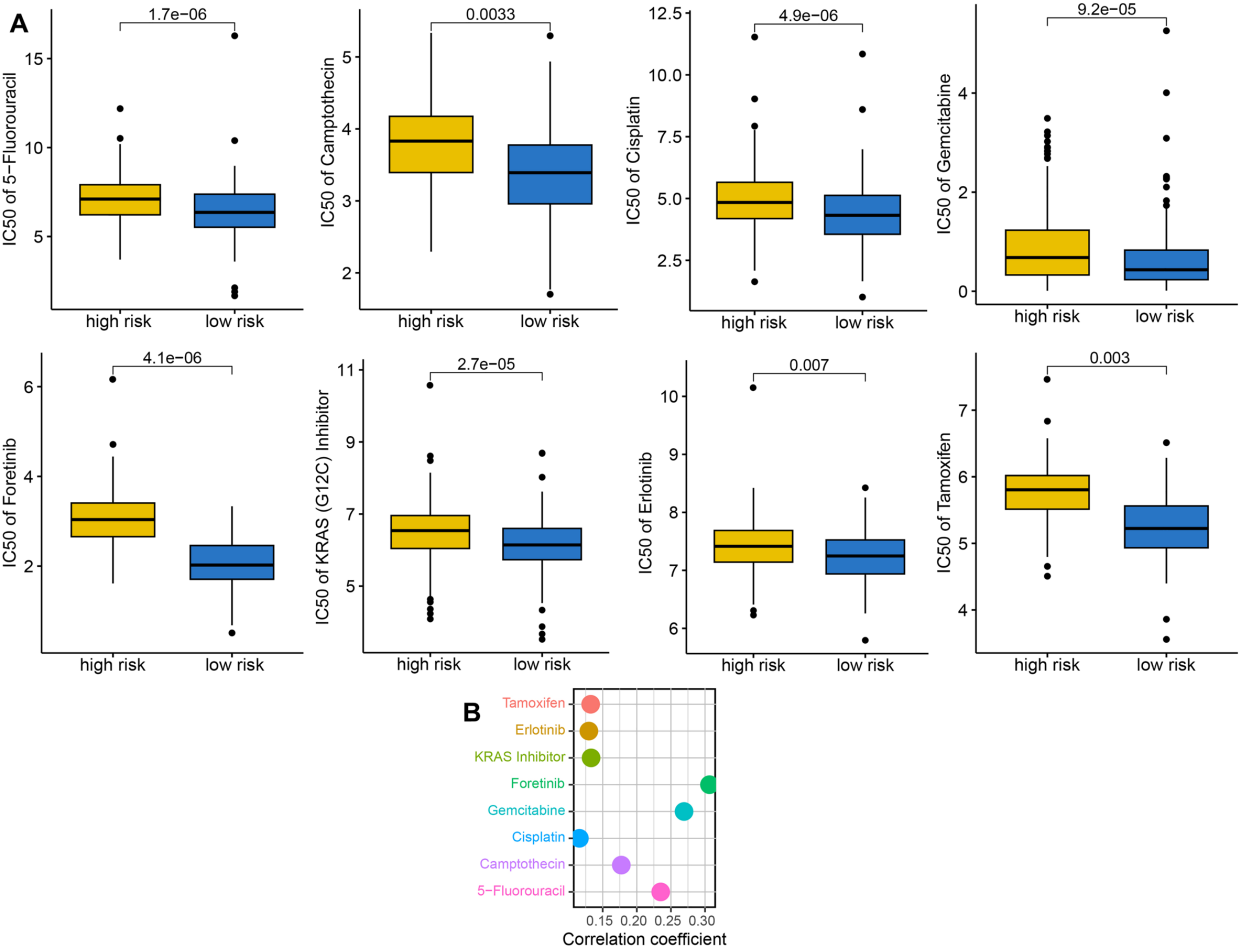
The IC50 value of common drugs in different TRPS score group. (**A**) Low risk score indicated a lower IC50 value of common drugs. (**B**) The correlation between IC50 value of common drugs and TRPS score.

High HLA-related gene expression indicated wider range of antigen presentation, increasing the likelihood of presenting more immunogenic antigens, and the likelihood of benefiting from immunotherapy^11^. We found that OC patients with low risk score had a higher HLA-related genes in TCGA dataset (Fig. 6A, all P < 0.05). Immune checkpoints played a vital role in immune escape of cancer. Based on our results, the expression of most of immune checkpoints was higher in high risk score groups in OC in TCGA dataset (Fig. 6B, all P < 0.05). Previous study showed that high TMB score was correlated with a better response to immunotherapy^12^. IPS was a superior predictor of response to anti-CTLA-4 and anti-PD-1 antibody and high IPS indicated a better response to immunotherapy^13^. High TIDE score indicated a greater likelihood of immune escape and less effectiveness of ICI treatment^14^. As showed in Fig. 6C–F, OC patients with low risk score had a higher TMB score, higher PD1 immunophenoscore, CTLA4 immunophenoscore, and PD1&CTLA4 immunophenoscore, lower immune escape score, lower TIDE score, lower T cell exclusion and dysfunction score in TCGA dataset. Thus, OC patients with low risk score may have a better immunotherapy benefit. To further verify the predictive value of TRPS in immunotherapy benefits, we then applied two immunotherapy cohorts to further verify our results. As shown in Fig. 6G, the risk score in non-responders was significantly higher than that in responders in IMvigor210 cohort (P < 0.01). Moreover, high risk score indicated a poor clinical outcome and lower response rate in IMvigor210 cohort (Fig. 6G). Similar results were obtained in GSE91061 cohort (Fig. 6H). As the vital role of chemotherapy, targeted therapy and endocrinotherapy for the treatment of OC, we also detected the IC50 value of common drugs in OC patients. We found that the IC50 value of 5-Fluorouracil, Camptothecin, Cisplatin, Gemcitabine, Foretunib, KRAS inhibitor, Erlotinib, and Tamoxifen were higher in in OC patients with high risk score in TCGA dataset (Fig. 7A, all P < 0.05). Moreover, positive correlation was obtained between risk score and these drugs in TCGA dataset (Fig. 7B). Thus, OC patients with low risk score may be better sensitivity to chemotherapy and targeted therapy.

### The distinct difference in cancer related hallmarks in OC patients with different TRPS score

**Figure 8:**
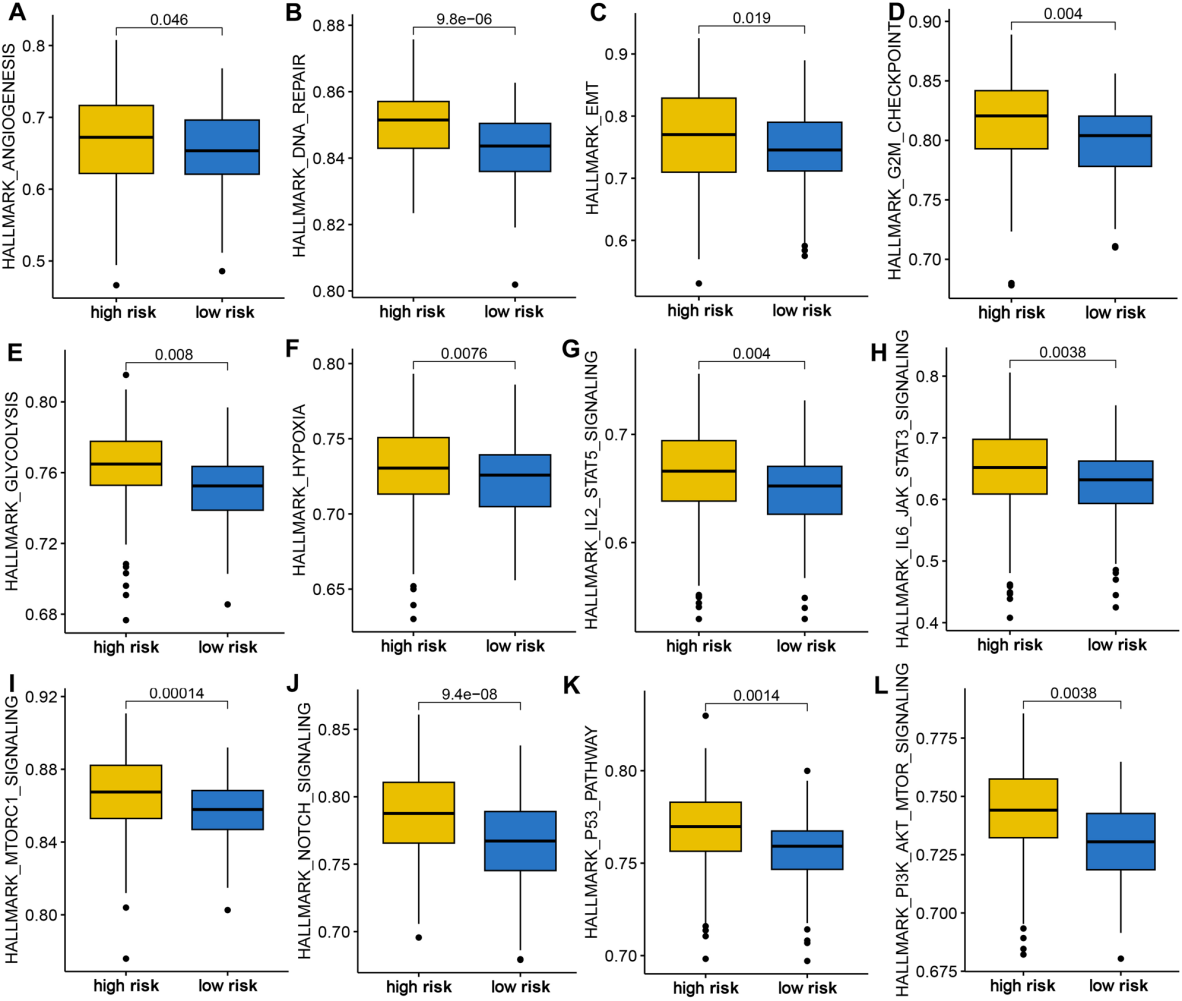
Gene set enrichment analysis in different TRPS score group. High risk score indicated a higher score of angiogenesis (**A**), DNA repair (**B**), EMT (**C**), G2M checkpoint (**D**), glycolysis (**E**), hypoxia (**F**), IL2-STAT5 signaling (**G**), IL6-JAK-STAT3 signaling (**H**), MTORC1 signaling (**I**), NOTCH signaling (**J**), P53 pathway (**K**), and P13K-AKT-mTOR signaling (**L**).

We finally performed gene set enrichment analysis to explore the potential mechanism mediating the difference of OC patients in clinical outcome, immune infiltration, and therapy response. High risk score indicated a higher sore of angiogenesis, DNA repair, EMT, G2M checkpoint, glycolysis, hypoxia, IL2-STAT5 signaling, IL6-JAK-STAT3 signaling, MTORC1 signaling, NOTCH signaling, P53 pathway, and P13K-AKT-mTOR signaling in OC in TCGA dataset (Fig. 8A–L, all P < 0.05).

### Biological functions of the selected gene

**Figure 9:**
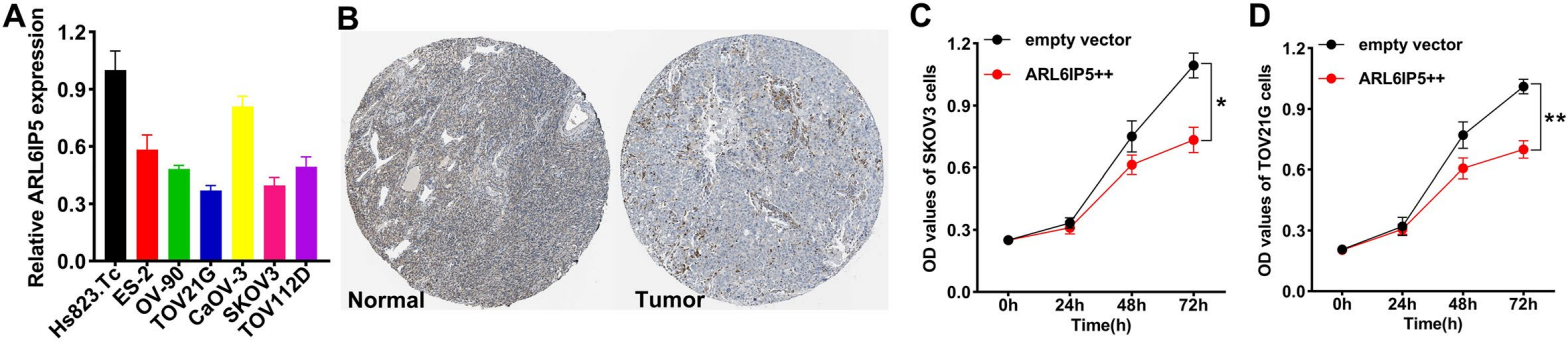
Validation of the potential function of ARL6IP5 in OC by in vitro assays. (**A**) Comparison of ARL6IP5 expressions in normal and OC cell lines. (**B**) Typical immunohistochemical of ARL6IP5 in OC and normal tissues. (**C**,**D**) CCK-8 assay showed that overexpression of ARL6IP5 obviously inhibited the proliferation of SKOV3 and TOV21G cells. *P < 0.05, **P < 0.01.

To further verify the performance of TRPS, we selected ARL6IP5 that contributed the most to the TRPS for further analysis. We first examined the expression of ARL6IP5 in OC cell lines, which showed that the expression of ARL6IP5 was lower in OC cell lines (Fig. 9A). Typical immunohistochemical of ARL6IP5 in OC and normal tissues were showed in Fig. 9B. In the follow-up study, the results of the CCK-8 assay proved that overexpression of ARL6IP5 obviously inhibited the proliferation of SKOV3 and TOV21G (Fig. 9C,D).

## Discussion

In our study, we developed a TRPS by using 10 integrative machine learning methods in TCGA dataset. The TRPS acted as an independent risk factor for OC and showed stable and powerful performance in predicting the clinical outcome of OC patients. Compared with clinical stage, and tumor grade, our TRPS had a higher C-index. These findings were also verified in GSE14764, GSE26193, GSE63885 and GSE140082 cohort. Further analysis showed that TRPS serve as an indicator for predicting the immune infiltration, immunotherapy benefits of OC patients.

The TRPS was developed based on 18 TRGs, including CXCL3, ALOX5A, CD3G, ETV7, ISG20, STAT1, BLOC1S1, NDUFV2, PSMA2, PSMA5, ZFP36L1, SERPINB1, KRAS, SPCS, ARL6IP5, GBP2, SRP9, FLEKHF1. Previous studies have showed that these genes played a vital role in the development of OC or other types of cancer. ETV7 could result in doxorubicin resistance by mediating DNAJC15 repression in breast cancer^15^. ISG20 promoted tumor progression in ccRCC and acted as a potential biomarker^16^. STAT1-induced upregulation lncRNA LINC00958 and promoted the tumorigenesis of OC via Wnt/β-Catenin signaling^17^. PSMA5 accelerated the tumorigenic process and involved in bortezomib resistance in prostate cancer^18^. ZFP36L1 accelerated tumor progression by mediating JNK and p38 MAPK signaling pathways in gastric cancer^19^.

Immunotherapy was one of the best treatment options for cancer patients with advanced disease^20,21^. Recent study highlighted the vital function of activation of anti-tumor immunity in eradicating tumor cells^22^. However, the evidence on the sensitivity of ovarian cancer to immunotherapy was still relatively limited, needing further exploration. High TIDE score indicated a greater likelihood of immune escape and less effectiveness of ICI treatment^14^. IPS was a superior predictor of response to anti-CTLA-4 and anti-PD-1 antibody and high IPS indicated a better response to immunotherapy^13^. High TMB score was correlated with a better response to immunotherapy^12^. OC patients with low risk score had a higher PD1&CTLA4 immunophenoscore, higher TMB score, higher HLA-related genes, lower TIDE score, lower tumor escape score and lower immune checkpoints expression, suggesting TRPS as an indicator for predicting immunotherapy benefit.

To explore the potential mechanism leading to the difference of different TRPS score in clinical outcome, immune infiltration, and therapy response, we then analyzed the cancer-related gene set score in different TRPS score group in OC. The data demonstrated that high TRPS score indicated higher score of angiogenesis, DNA repair, EMT, glycolysis, hypoxia, IL2-STAT5 signaling, IL6-JAK-STAT3 signaling, NOTCH signaling, P53 pathway, and P13K-AKT-mTOR signaling. These signaling played a vital role in the development and immune response of OC. Angiogenesis acted as therapeutic targets in OC and involved in tumor metastasis^23^. Glycolysis was correlated with chemoresistance and T cell function in OC^24,25^. Previous study also highlighted the vital role of NOTCH signaling immune responses and tumor progression of OC^26^. Moreover, hypoxia in the microenvironment could affect the immunotherapy outcome of OC^27^.

Some limitations and shortcomings remain in our study. The expression and prognosis of TRPS genes should be verified by using clinical tissues. Moreover, it would be better to explore the mechanism of TRPS in the progression of OC.

## Conclusion

The current study constructed a novel TRPS for OC, which could serve as an indicator for predicting the prognosis, immune infiltration and immunotherapy benefits of OC patients.

### Supplementary Information

Supplementary Information.

Supplementary Table 1.

## Data Availability

The datasets utilized in this study are available in TCGA (TCGA-BLCA, https://portal.gdc.cancer.gov/) and GEO repository (GSE14764, GSE26193, GSE26712, GSE63885 and GSE140082, https://www.ncbi.nlm.nih.gov/geo/).
