# Supplementary material for: Machine learning developed a CD8+ exhausted T cells signature for predicting prognosis, immune infiltration and drug sensitivity in ovarian cancer
Source: Sci Rep. 2024 Mar 9;14:5794. doi: 10.1038/s41598-024-55919-4 (PMC10925064; doi:10.1038/s41598-024-55919-4)
Supplement: Supplementary file 1 — Supplementary Information. [file 41598_2024_55919_MOESM1_ESM.docx]

**scRNA-seq analysis**

scRNA-seq data was processed with the Seurat R package (version 4.0) [1]. Those genes detected in more than 3 cells, cells with more than 200 detected genes or cells with a mitochondrial proportion of less than 20% would be select for further analysis. Cell populations on a two-dimensional map was determined by principal component analysis and tSNE analysis. Pseudotime analysis. An analysis of pseudotime, also known as cell trajectory analysis, helps predict the evolutionary trajectory of apoptosis pathways and cell subtypes and infer the differentiation trajectory of stem cells during disease progression. By analyzing key gene expression patterns using Monocle 2, we performed pseudotime analysis in T/K cells in the current study. The pseudotime value was used by monocle to model the gene expression level as a nonlinear smooth pseudotime function to show change in gene expression with time.

**Machine learning algorithms developed a prognostic TRPS**

A total of 10 machine learning methods were included in the procedure, including random survival forest (RSF), elastic network (Enet), Lasso, Ridge, stepwise Cox, CoxBoost, partial least squares regression for Cox (plsRcox), supervised principal components (SuperPC), generalized boosted regression modelling (GBM), and survival support vector machine (survival-SVM). Based on the process of the R scripts (https://github.com/Zaoqu-Liu/IRLS) of previous study[2, 3], we constructed the TRPS using following steps: (1) Univariate Cox regression was conducted to identify prognostic biomarkers in the TCGA dataset; (2) Then, the prediction model of TCGA data set is fitted with 90 algorithms combinations; (3) All algorithms combinations were performed in GEO cohorts; (4) C-index was calculated across all cohorts.

**Immune infiltration analysis**

Immunedeconv, an R package integrating 7 state-of-the-art algorithms (CIBERSORT, MCPcounter, QUANTISEQ, XCELL, CIBERSORT-ABS, TIMER and EPIC) was used to explore the correlation between risk score and immune cells. To evaluate to the immune and ESTIMATE score of each OC case, we then applied “estimate” R package [4].

**Integrative machine learning algorithms developed a TRPS.**

**Risk score = (-0.0213)×CXCL3^exp^ + 0.1438×ALOX5AP^exp^ + (-0.1396)×CD3G^exp^ + (-0.0516)×ETV7^exp^ + (-0.0046)×ISG20^exp^ + (-0.1623)×STAT1^exp^ + (-0.1558)×BLOC1S1^exp^ + (-0.0618)×NDUFV2^exp^ + (-0.0369)×PSMA2^exp^ + (-0.0674)×PSMA5^exp^ + 0.0257×ZFP36L1^exp^ + (-0.0999)×SERPINB1^exp^ + 0.1212×KRAS^exp^ + (-0.0126)×SPCS^exp^ + 0.1790×ARL6IP5^exp^ + (-0.0892)×GBP2^exp^ + (-0.1031)×SRP9^exp^ + (-0.0390)×FLEKHF1^exp^.**

**Reference**

1. Sun Y, Wu L, Zhong Y, Zhou K, Hou Y, Wang Z, Zhang Z, Xie J, Wang C, Chen D *et al*: **Single-cell landscape of the ecosystem in early-relapse hepatocellular carcinoma**. *Cell* 2021, **184**(2):404-421.e416.

2. Liu Z, Liu L, Weng S, Guo C, Dang Q, Xu H, Wang L, Lu T, Zhang Y, Sun Z *et al*: **Machine learning-based integration develops an immune-derived lncRNA signature for improving outcomes in colorectal cancer**. *Nature communications* 2022, **13**(1):816.

3. Li Z, Guo M, Lin W, Huang P: **Machine Learning-Based Integration Develops a Macrophage-Related Index for Predicting Prognosis and Immunotherapy Response in Lung Adenocarcinoma**. *Archives of medical research* 2023, **54**(7):102897.

4. Yoshihara K, Shahmoradgoli M, Martínez E, Vegesna R, Kim H, Torres-Garcia W, Treviño V, Shen H, Laird PW, Levine DA *et al*: **Inferring tumour purity and stromal and immune cell admixture from expression data**. *Nat Commun* 2013, **4**:2612.
